# Pathologic Prognostic Factors in Endometrial Carcinoma (Other Than Tumor Type and Grade)

**DOI:** 10.1097/PGP.0000000000000524

**Published:** 2018-12-14

**Authors:** Naveena Singh, Lynn Hirschowitz, Richard Zaino, Isabel Alvarado-Cabrero, Maire A. Duggan, Rouba Ali-Fehmi, Elizabeth Euscher, Jonathan L. Hecht, Lars-Christian Horn, Olga Ioffe, Xavier Matias-Guiu, W. Glenn McCluggage, Yoshiki Mikami, Jaume Ordi, Vinita Parkash, M. Ruhul Quddus, Charles M. Quick, Annette Staebler, Charles Zaloudek, Marisa Nucci, Anais Malpica, Esther Oliva

**Affiliations:** Department of Cellular Pathology, Barts Health NHS Trust, London (N.S.); Department of Cellular Pathology, Birmingham Women’s NHS Trust, Birmingham (L.H.); Department of Pathology, Belfast Health and Social Care Trust, Belfast (W.G.M.), UK; Division of Anatomic Pathology, Hershey Medical Center, Pennsylvania State University, Hershey, Pennsylvania (R.Z.); Department of Pathology, Mexico City Hospital of Oncology, Mexico City, Mexico (I.A.-C.); Department of Pathology and Laboratory Medicine, University of Calgary, Calgary, Alberta, Canada (M.A.D.); Department of Pathology, Wayne State University, Detroit, Michigan (R.A.-F.); Department of Pathology, The University of Texas MD Anderson Cancer Center, Houston, Texas (E.E., A.M.); Department of Pathology, Beth Israel Deaconess Medical Center and Harvard Medical School (J.L.H.); Department of Pathology, Brigham and Women’s Hospital, Harvard Medical School (M.N.); Department of Pathology, Massachusetts General Hospital, Harvard Medical School (E.O.), Boston, Massachusetts; Division of Gynecologic, Breast & Perinatal Pathology, University Hospital Leipzig, Leipzig (L.-C.H.); Institute of Pathology, University Hospital of Tübingen, Tübingen (A.S.), Germany; Department of Anatomical Pathology, University of Maryland, College Park, Maryland (O.I.); Pathological Oncology Group and Pathology Department, University Hospital of Arnau de Vilanova, Lleida (X.M.-G.); Department of Pathology, Hospital Clinic of Barcelona, ISGlobal, Barcelona Center for International Health Research, University of Barcelona, Barcelona (J.O.), Spain; Department of Diagnostic Pathology, Kumamoto University Hospital, Kumamoto, Japan (Y.M.); Department of Pathology, Yale University School of Medicine, New Haven, Connecticut (V.P.); Department of Pathology, Women and Infants Hospital/Warren Alpert Medical School of Brown University, Providence, Rhode Island (M.R.Q.); Department of Pathology, University of Arkansas for Medical Sciences, Little Rock, Arkansas (C.M.Q.); Department of Pathology, University of California, San Francisco, San Francisco, California (C.Z.)

**Keywords:** Endometrial carcinoma, Prognosis, Pathology, Myometrium invasion, MELF, Cervical stromal invasion, Lymphovascular space invasion, Adnexal involvement, Staging

## Abstract

Although endometrial carcinoma (EC) is generally considered to have a good prognosis, over 20% of women with EC die of their disease, with a projected increase in both incidence and mortality over the next few decades. The aim of accurate prognostication is to ensure that patients receive optimal treatment and are neither overtreated nor undertreated, thereby improving patient outcomes overall. Patients with EC can be categorized into prognostic risk groups based on clinicopathologic findings. Other than tumor type and grade, groupings and recommended management algorithms may take into account age, body mass index, stage, and presence of lymphovascular space invasion. The molecular classification of EC that has emerged from the Cancer Genome Atlas (TCGA) study provides additional, potentially superior, prognostic information to traditional histologic typing and grading. This classifier does not, however, replace clinicopathologic risk assessment based on parameters other than histotype and grade. It is envisaged that molecular and clinicopathologic prognostic grouping systems will work better together than either alone. Thus, while tumor typing and grading may be superseded by a classification based on underlying genomic abnormalities, accurate assessment of other pathologic parameters will continue to be key to patient management. These include those factors related to staging, such as depth of myometrial invasion, cervical, vaginal, serosal surface, adnexal and parametrial invasion, and those independent of stage such as lymphovascular space invasion. Other prognostic parameters will also be discussed. These recommendations were developed from the International Society of Gynecological Pathologists Endometrial Carcinoma project.

Endometrial carcinoma (EC) is generally considered to be associated with a good prognosis, largely because low-grade endometrioid carcinoma is the most common subtype and is typically low stage at clinical presentation. However, over 20% of women with EC die of their disease, and there is a projected increase in both incidence and mortality over the next few decades. The aim of accurate and reproducible prognostication is to ensure that patients receive optimal treatment and are neither overtreated nor undertreated, thereby improving patient outcomes overall.

Histologic parameters that need to be recorded in hysterectomy specimens according to the recommendations of the National Comprehensive Cancer Network are listed in Table 1 1. Other than tumor type and grade, prognostic risk groupings and recommended management algorithms take into account stage, as well as age, fitness for surgical treatment, fertility-conservation, presence of lymphovascular space invasion (LVSI), and other factors. Recommendations show minor variation in different jurisdictions and Tables 2–5 list the most widely followed National Comprehensive Cancer Network and European Society for Medical Oncology presurgical and postsurgical recommendations; it should be noted that the latter, that is recommendations for adjuvant treatment, are made almost entirely on the basis of pathologic findings 1–3.

**TABLE 1:**
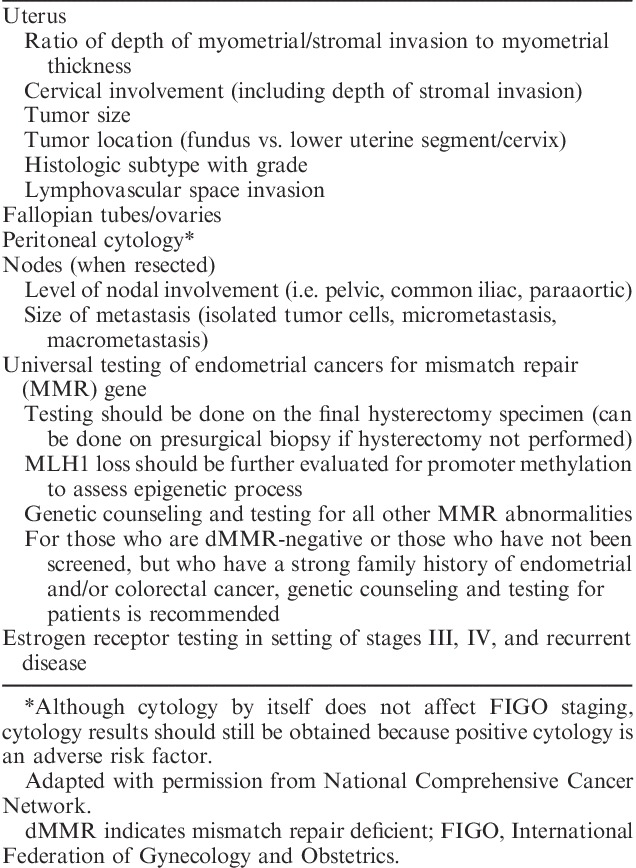
Pathologic assessment to be included in evaluation of hysterectomy specimens 1

**TABLE 2:**
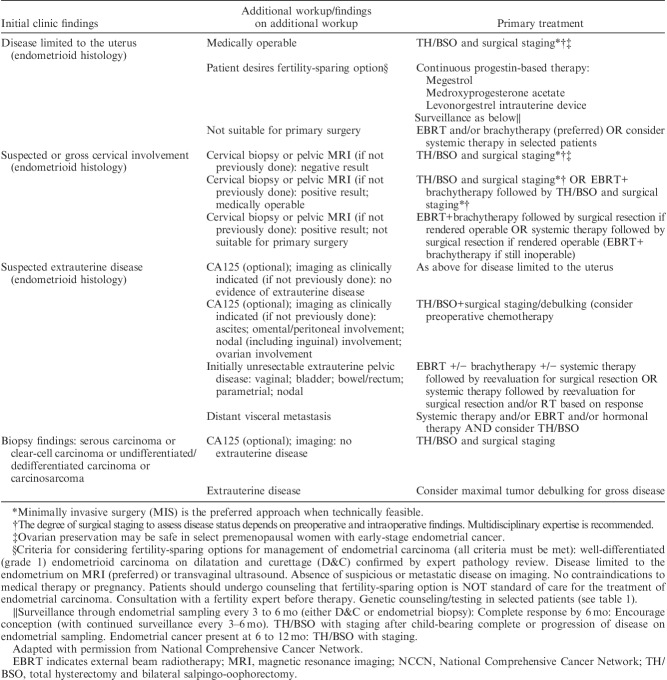
NCCN endometrial cancer primary treatment algorithms 1

**TABLE 3:**
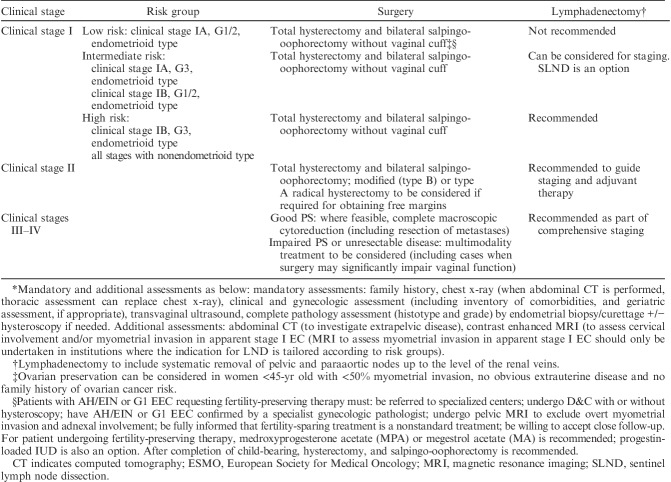
ESMO endometrial cancer surgical management algorithms based on preoperative assessment* 1,2

**TABLE 4:**
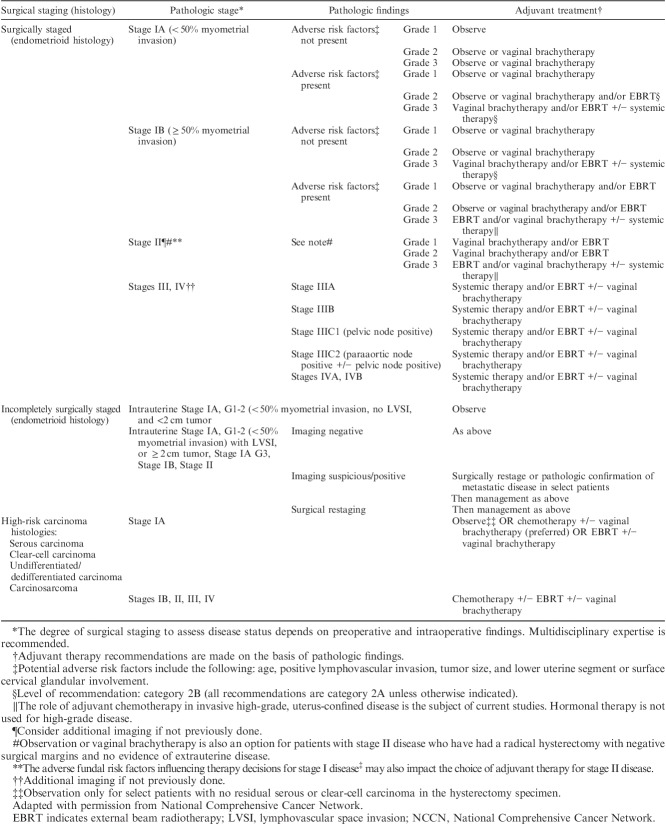
NCCN adjuvant therapy determinations for endometrial carcinoma based on pathologic findings 1

**TABLE 5:**
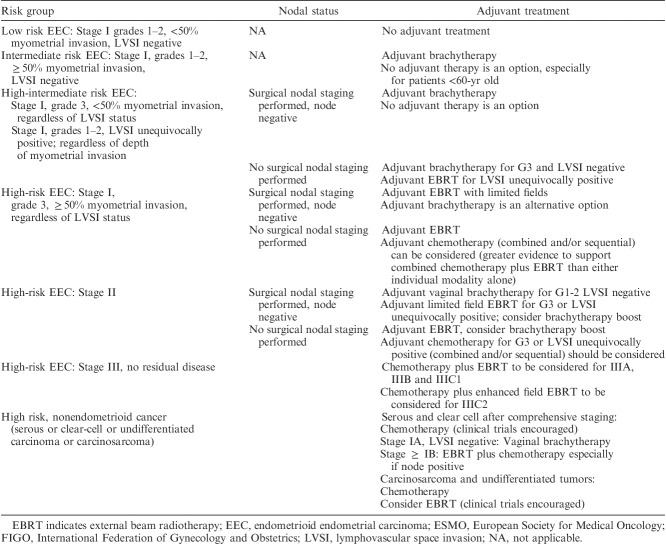
ESMO endometrial cancer adjuvant treatment algorithm based on final histotype and postsurgical staging according to FIGO 2009 system 1,2

The molecular classification of EC that has emerged from the Cancer Genome Atlas (TCGA) study provides additional potentially superior prognostic information to traditional histologic typing and grading. This classifier does not, however, replace clinicopathologic risk assessment based on parameters other than histotype and grade 4–6. It is envisaged that molecular and clinicopathologic prognostic grouping systems will likely work better together. Thus, while tumor typing and grading may be partly or totally superseded by a classification based on underlying genomic abnormalities, accurate assessment of pathologic parameters other than tumor morphology will continue to be key to patient management. Lack of agreement in the assessment of these parameters is well documented in the literature 7–12 and this article focuses on ways to minimize disagreement and promote uniformity in the approach to their recognition. These include those factors that are related to staging 13,14 (Table 6), and those independent of stage.

**TABLE 6:**
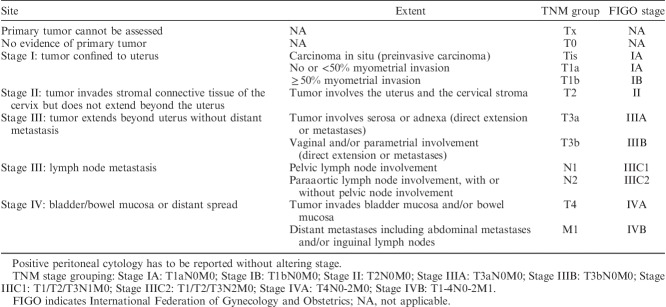
FIGO and TNM staging systems of endometrial carcinoma

These recommendations were developed from the International Society of Gynecological Pathologists Endometrial Carcinoma project.

## PATHOLOGIC PROGNOSTIC FACTORS

### Tumor Stage

Recommendations:

- Provisional pathologic staging should be provided in the pathology report for all hysterectomy specimens from cases of EC.
- The TNM staging system (Union for International Cancer Control and American Joint Committee on Cancer versions) for EC is largely concordant with the widely used International Federation of Gynecology and Obstetrics (FIGO) system (Table 6) 15. Regardless of the system used, staging remains the most powerful prognostic indicator in EC and depends on accurate assessment of a range of pathologic factors that are individually considered below.
- Pathologic reporting guidelines worldwide 16 recommend that tumor stage should be provided in the pathology report as FIGO stage, and in some jurisdictions TNM staging is also mandated, specifying the version used. The International Collaboration on Cancer Reporting (ICCR) and Royal College of Pathologists (UK) (RCPath) datasets stress that staging in the pathology report is provisional as the final stage should be determined when all relevant clinical and radiologic information is available to be integrated with the pathologic findings.
- Some locations of tumor spread are not specifically addressed in the FIGO staging system. A few that are listed below have been confirmed by the TNM helpdesk (Dr Lynn Hirschowitz, personal communication, written form):
- Spread to pelvic serosa including bladder, sigmoid serosa and cul de sac is staged as FIGO IIIA.
- Spread to serosa in the abdominal cavity (abdominal peritoneal involvement) is staged as FIGO IVB (pM1).
- Spread to omentum (supracolic or infracolic) is staged as FIGO IVB (pM1).

It should be noted that, for the purposes of staging gynecologic cancers, pelvic peritoneum (and organs within pelvis) are within the “true pelvis,” and abdominal peritoneum and related organs are outside the confines of the true pelvis. The true pelvis, also known as the lesser pelvis, is below the level of the pelvic brim, between the pelvic inlet and the pelvic floor 17. The true pelvis contains the sigmoid colon, rectum, and bladder, as well as all gynecologic organs.

### Myometrial Invasion

Recommendations:

- Absence or presence and depth of myometrial invasion should be reported in all EC as “none or less than half” OR “half or more.”

- Percentage invasion or measurements of depth and total myometrial thickness from which the above can be derived are acceptable alternatives.

- If myometrial invasion occurs from carcinoma within adenomyosis, the deepest myoinvasive point should be reported according to where this is located in the myometrium, and regardless of whether or not it arises from adenomyosis.

- In low-grade endometrioid EC where invasion arising from adenomyotic foci in the outer half of the myometrium is the only focus of invasion in the outer half, this should be noted and accompanied by a comment that the clinical significance is unknown, and that this may be an overestimate of true depth of invasion. In other words, in the absence of tumor extension to other organs, the tumor should be staged as FIGO IB, with a proviso that it could be an overestimation of the actual depth of invasion.

- In case of an exophytic tumor, the depth of myometrial invasion, and not tumor thickness, should be measured by identifying the adjacent endomyometrial junction and by correlating with the macroscopic appearance.
- For tumors involving polyps, measurement of invasion is performed only if the tumor invades the underlying myometrium and measurement should be performed from the adjacent endometrial-myometrial interface. Invasion into polyp stroma can be described, but its depth should not be measured.
- The depth of myometrial invasion of tumors within the lower uterine segment should be measured as elsewhere in the uterine corpus; that is, as “none or less than half” OR “half or more” of myometrial thickness at that location.
- When EC invades a leiomyoma, the thickness of the myometrial wall at this site should be measured to include the leiomyoma and this distance should be used in calculating the depth of invasion.
- In EC with a microcystic, elongated and fragmented (MELF) pattern of invasion, the presence of desmoplasia alone is insufficient to measure depth of invasion. Levels and cytokeratin stains may be examined if necessary to identify malignant cells within these areas and measurement of deepest invasion should take into account the deepest area where these are seen.
- LVSI should not be included in assessment of myoinvasive depth; only carcinoma infiltrating the myometrium is included in this measurement.

Depth of myometrial invasion has consistently been found to be an independent predictor of both lymph node (LN) metastasis and overall prognosis in EC. For this reason, depth of invasion has been a component of the FIGO staging system for EC for over 2 decades. However, depth of myometrial invasion is not always easy to assess as the endometrium has an irregular interface with the underlying myometrium and it is reported that pathologists disagree on myoinvasive depth in about 30% of cases 11. Measurement of myoinvasion is performed only if the tumor invades the myometrium in relation to the endometrial-myometrial interface. The demarcation zone is the midpoint of an axis drawn from the external surface of the uterus to the junction of the closest non-neoplastic endometrial lining and underlying myometrium. Depth of myometrial invasion is assessed as the deepest point of invasion relative to the demarcation zone, and expressed as “less than half” or “half or more.” When the junction is completely obliterated, or there is no junction from which to measure, the vascular arcuate plexus can be used to assess myoinvasive depth; invasion well into or through the plexus usually signifies >50% myoinvasion; however, if infiltration barely extends into the plexus, the depth of invasion should be calculated with reference to the thickness of the myometrium in the opposite uterine wall 11,18. It should be noted that LVSI is not included in assessment of myoinvasive depth; only carcinoma infiltrating the myometrium is included in this measurement.

In some instances, assessment of depth of myometrial invasion can be particularly challenging, such as with exophytic tumors, or those involving polyps, or in areas where the myometrium is thin such as in the uterine cornua or isthmus, or when invasion emanates from adenomyosis or involves a leiomyoma. A further challenge is whether or not to include desmoplasia without evident neoplastic cells in tumors showing a MELF pattern of invasion.

#### Tumors With an Exophytic Growth or Involving Polyps

In the first scenario, it is important to note that exophytic tumors often incorporate smooth muscle within their core and this should not be misinterpreted as myometrial invasion. Thus, depth of invasion but not tumor thickness should be measured by identifying the adjacent endomyometrial junction and correlating the findings with the macroscopic location of the tumor 11. Similarly, for tumors involving polyps, invasion into the polyp stroma may occur but this does not represent myometrial invasion. Measurement of myoinvasion is performed only if the tumor invades the myometrium in relation to the endometrial-myometrial interface. Correlation with gross findings is useful, as well as taking relevant sections from elsewhere in the uterine wall, that is, not only those sections that represent the tumor either in its exophytic areas or within the polyp.

#### Myometrial Invasion From a Focus of Adenomyosis

Several studies have shown that ECs confined to foci of adenomyosis have a prognosis that is similar to those confined to the endometrium; involvement confined to adenomyotic foci should therefore not be misinterpreted as evidence of myometrial invasion. Rarely, there may be myometrial invasion only at a site where tumor involves adenomyosis. There are 2 possible ways to address depth of invasion in this scenario:

1. Assess the deepest point of invasion based on where it is located in the myometrium regardless of whether or not it from adenomyosis, in relation to the myometrial thickness as assessed above 19.
2. Measure from the edge of the adenomyotic focus to the furthest point of invasion 20, and express this as a fraction of myometrial thickness.

In deciding which of these 2 approaches to recommend, we reviewed the impact of the presence of adenomyosis on prognosis in patients with EC, as there are no data directly comparing these 2 methods of assessing depth of invasion associated with adenomyosis to determine which is more prognostically discriminatory. In some studies, the presence of adenomyosis in low-grade endometrioid carcinoma has no impact on prognosis 21,22 while some regard it as a marker of less aggressive disease 23,24. In other studies, the presence of adenomyosis is associated with a higher incidence of myometrial invasion, likely due to increase in tumor interface surface area with the adjacent myometrium, as well as a slightly increased likelihood of deeper myometrial invasion; however, this finding does not appear to impact on overall prognosis as typically foci of invasion appear to be small when compared with deeply invasive EC without associated adenomyosis 22,25–27.

**FIG. 1:**
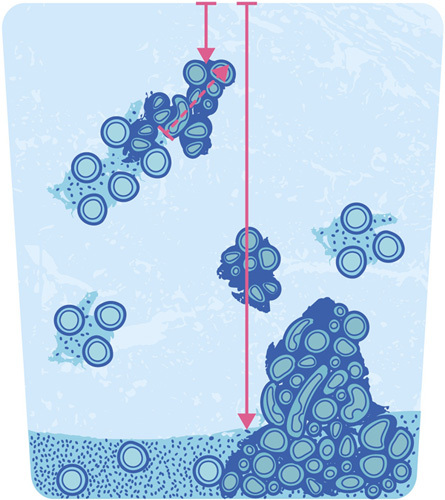
If myometrial invasion occurs from carcinoma within adenomyosis, the distance of the deepest myoinvasive point from the outer surface of the myometrium (short solid arrow) should be measured in relation to the myometrial thickness (long solid arrow), rather than the distance of the invasive depth of the focus (dashed arrow) taken as the extent of myoinvasion. Courtesy of Lucas Catalan Galan and Laura Casey.

In the absence of randomized trial data with direct comparison of outcomes based on these 2 methods of assessment of depth of invasion of tumor arising from adenomyosis, the group felt it to be preferable to adhere to standard and reproducible protocols. Of the 2 approaches listed, the first is more likely to be reproducible and less error-prone, whereas the second approach presents a possible danger of understaging an EC with deep myoinvasion. In the latter setting, interobserver variability can be anticipated given the absence of established measurement criteria. Furthermore, accurate measurement of depth of invasion may be difficult as the orientation of the adenomyotic foci may differ from the normal endomyometrial plane. Thus, it is preferable to use the standard method for determining depth of invasion, based on the location of the deepest focus of invasive carcinoma in relation to the total myometrial thickness in this area, irrespective of its relationship to adenomyosis (Fig. 1). In cases where the EC appears to be directly invading from adenomyosis, this finding should be commented on in the report, as it may be associated with less aggressive behavior than that of tumors with conventional invasion at a similar depth. It is acknowledged by the authors that this is an area that requires further research.

#### Myometrial Invasion in Cornual or Lower Uterine Segment/Isthmic (LUS) Locations

Literature is scant in providing guidelines to measure depth of invasion for tumors located in the LUS or cornua. It is important to note that these regions have a much thinner myometrium. The LUS is easy to identify from sections that contain the inactive or ciliated glands and fibrous stroma characteristic of this location or also contain upper endocervix or from the section code but location in the cornu may be overlooked when grossing. In general, it is agreed that depth of invasion should be measured as elsewhere in the uterine corpus but it is recommended that sections from the cornual region should not be used to determine myoinvasive depth unless the tumor is located wholly in this region or it reaches/breaches the serosa only in this region 28.

#### Tumors With MELF Pattern of Invasion

**FIG. 2:**
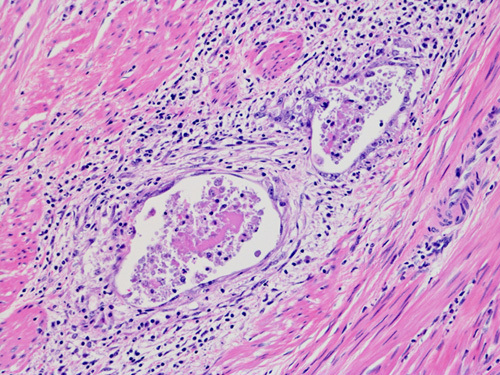
Infiltrative glands in foci showing a microcystic, elongated, and fragmented pattern may be difficult to appreciate, as the lining is attenuated and individual tumor cells may have a histiocytoid appearance.

A MELF pattern of myometrial invasion may be associated in the deepest areas with a desmoplastic response or inflammatory reaction which should not be counted as the deepest point of invasion if no associated malignant cells are identified. Especially in inflammatory areas, malignant cells may be difficult to appreciate as they can display a histiocytoid morphology with very innocuous cytologic features that may result in misinterpretation as true histiocytes (Fig. 2). In either scenario, if malignant cells are not seen, levels and/or cytokeratin staining can be performed, though the latter are not generally recommended, or extra sections may be taken to determine myoinvasive depth 29–32.

### Cervical Involvement

Recommendations:

- Presence or absence of cervical stromal invasion must be recorded as its presence upstages an EC to stage II.
- Recognition of cervical stromal invasion may be facilitated by the finding of a desmoplastic/inflammatory reaction but may occur in its absence. In the latter situation, recognition is facilitated by the observation of altered architecture relative to normal endocervical crypts.
- For the purposes of standard reporting, the uppermost endocervical mucinous gland identified in the section should be taken as the upper limit of the endocervix.
- In the presence of cervical stromal invasion, the status of radial and distal margins (including minimum distance) should be included as well as depth of invasion within the cervical stroma.
- Cervical epithelial involvement is no longer part of FIGO staging for EC; however, it is recommended that this finding should be included in the report.

Historically, cervical involvement has been considered important for prognostication in patients with EC. However, since 2009, involvement limited to cervical epithelium is no longer considered as FIGO stage II (previously as IIA) as it has been shown that patients with cervical epithelial involvement have identical outcomes to those with stage I tumors. However, this is still included as a potential adverse risk factor for consideration of adjuvant therapy 1. As patients with EC associated with cervical stromal invasion have a worse prognosis (see below), this is categorized as FIGO stage II with a recommendation for adjuvant treatment 2.

Patients with tumors showing cervical involvement are considered to have a worse prognosis (overall survival) that those with tumors confined to the corpus. However, such patients generally have other known poor prognostic factors such as high tumor grade, deep myometrial invasion, and LVSI 33,34. When matched for other prognostic factors (cell type, grade, depth of myometrial invasion, LVSI, age, and nodal involvement), cervical involvement, including depth of cervical stromal invasion was not a significant prognosticator by univariate or multivariate analysis in a large study of 200 ECs 35. In another study, distinction between stages IIA and IIB (prior FIGO staging system) or depth of stromal invasion did not affect survival in patients with endometrioid EC. Only age, LVSI, and type of treatment were predictors of survival in patients with stage II endometrioid EC 36.

Cervical stromal invasion has also been correlated with recurrence in patients with EC. In 1 study, within the category of low-grade endometrioid ECs, univariate analysis showed cervical stromal involvement along with large tumor size, deep myoinvasion, MELF pattern of invasion, tumor necrosis, LVSI, and pelvic/paraaortic LN metastasis to be more common in patients whose tumors recurred at extravaginal sites 37.

Besides controversies regarding the prognostic significance of cervical involvement, including stromal invasion, reproducibility/accuracy in determining the presence and type of involvement has been shown to be low among pathologists 7,12,35. This diagnostic performance is related to a variety of morphologic issues that complicate the interpretation of cervical involvement in EC and which need to be addressed in order to standardize criteria, and increase accuracy and reproducibility 19. These include:

1. Boundaries between lower uterine segment and upper endocervix are poorly defined.
2. Florid reactive changes within endocervical glands may mimic secondary involvement by EC.
3. Tumor may be superficially implanted within cervical surface epithelium, sometimes in association with granulation tissue related to prior endometrial sampling.
4. Distinction of endocervical gland involvement from stromal invasion may be challenging and FIGO has not provided working definitions to distinguish gland from stromal involvement.
5. Some well-differentiated endometrioid ECs are not accompanied by a stromal response when infiltrating the cervix 38.

**FIG. 3:**
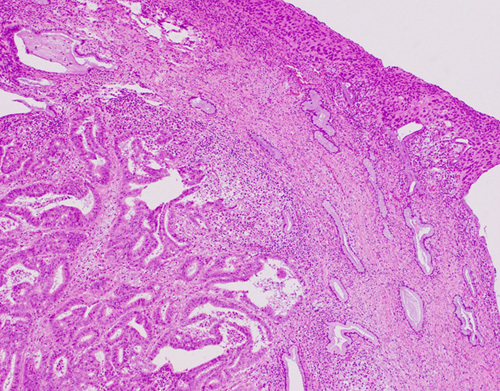
Cervical stromal involvement should be diagnosed when tumor shows confluent growth within the cervix or neoplastic glands are noted adjacent to preexisting endocervical glands.

Practice-based suggestions have been provided by the working group on how to recognize cervical stromal invasion. Cervical stromal invasion can be identified by the presence of a desmoplastic stromal response, and/or loss of the “normal” architectural arrangement of neoplastic glands in comparison to preexisting endocervical glands. In the latter scenario, the normal architecture of glands in uninvolved areas of endocervix should be utilized to assess preexisting endocervical architecture. Although no clear boundary exists, it is suggested that the uppermost mucinous endocervical gland be taken as the boundary between the LUS and endocervix but this may be challenging, as this area not infrequently has alternating mucinous and inactive/ciliated LUS glands. It is also recommended that endocervical stromal involvement be diagnosed when tumor shows a confluent growth within the cervix or neoplastic glands are noted in between preexisting endocervical glands 19 (Fig. 3).

In addition to identifying the presence of cervical stromal invasion, depth of invasion within the cervical wall should be reported 1. It may be a measurement in millimeters from the surface compared with the thickness of the cervical wall or as an indication of the proportion of the thickness of the cervical wall. In cases showing cervical stromal infiltration, the margin status should be reported as positive or negative (with minimum distance), for both the radial/outer cervical and distal margins 16,39.

### Uterine Serosal Involvement

Recommendations:

- Presence or absence of uterine serosal involvement should be recorded as its presence upstages an EC to stage IIIA.
- Tumor infiltrating the full myometrial thickness and reaching submesothelial fibroconnective tissue or the mesothelial layer should be reported as serosal involvement; tumor may or may not be present on the surface of the uterus; a desmoplastic response may or may not be present.

**FIG. 4:**
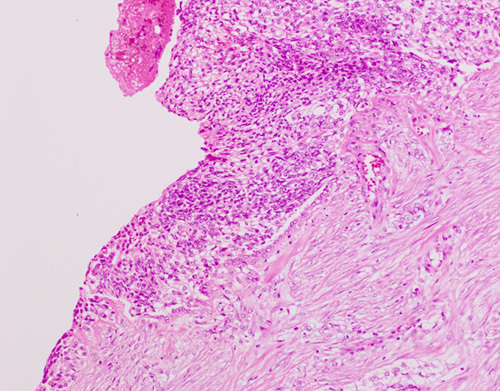
Tumor involving uterine serosal surface.

**FIG. 5:**
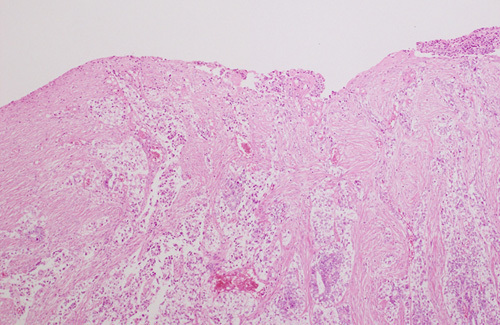
Serosal involvement may be accompanied by a desmoplastic and inflammatory reaction; the presence of tumor cells on the outer surface may not be obvious in individual sections. When tumor infiltrates the full myometrial thickness and involves the submesothelial fibroconnective tissue, this should be taken as evidence of serosal involvement.

There is essentially no information in the literature pertaining to the recognition of this parameter. For colorectal cancers, serosal involvement has been defined as “transgression of the serosal surface (i.e. mesothelial layer) by malignant cells” 40. The definition utilized for colorectal cancers may not apply to uterine cancers since the serosal layer investing the uterus is thin, consisting only of very scant fibroconnective tissue and mesothelium overlying a distinct and regular outer myometrial border, and there is no defined “subserosal” layer of adipose and fibroconnective tissue. As infiltrating ECs often elicit a desmoplastic response, tumor involving the serosa may be associated with collagenous stroma and the mesothelial layer may be imperceptible (as occurs frequently). Thus, the presence of tumor on the serosal surface may be obvious in most instances (Fig. 4), but sometimes difficult to recognize, even when multiple levels are examined. Uterine serosal involvement should therefore be defined as tumor infiltrating through the entire thickness of the myometrium and reaching the fibroconnective tissue or mesothelium, regardless of whether this appears to be exposed or not (Fig. 5). This is often associated with pallor, dullness, or discoloration of the outer uterine surface on gross examination and such areas should be submitted for histologic examination. Of note, the presence of lymphovascular space involvement within the serosa should not be considered as true serosal involvement or stage IIIA.

### Adnexal Involvement

Recommendations:

- The presence or absence of adnexal involvement should be recorded as its presence upstages an EC to stage IIIA.
- Care should be taken to determine whether the adnexal involvement is considered to be metastatic or “synchronous.”
- Type of adnexal involvement (ovarian parenchyma/surface, tubal mucosa/wall, paraadnexal tissue, or lymphovascular space involvement) should be recorded.
- The presence of lymphovascular space involvement only at any adnexal site does not affect stage.

Detailed histologic studies of the adnexal tissues in recent years have revealed that the issue of adnexal involvement by EC is more complicated than previously realized. ECs involving the adnexa are categorized as stage IIIA, and patients have an overall survival rate of ∼75%. This scenario has to be distinguished from synchronous primary tumors in the endometrium and the ovary or tube, that often are associated with an indolent outcome 41. To further complicate matters, recent studies have demonstrated a clonal relationship between some of these so-called independent synchronous tumors, especially endometrioid carcinomas 42,43. For these reasons, accurate classification of an adnexal neoplasm in a woman with endometrioid EC as metastatic versus synchronous may be a difficult task for the pathologist. The distinction between so-called synchronous independent adnexal and ECs and metastatic disease from one site to the other is discussed in more detail in another review in this issue 44.

Fallopian Tube Involvement—Recommendations:

- The presence of detached tumor cells in the tubal lumen should be reported, but this finding *per se* does not upstage EC.
- Tubal intraepithelial (mucosal) carcinoma, with or without stromal invasion, should, in the setting of a tumor within the endometrium, be viewed as metastasis from the EC, which should be upstaged due to tubal involvement.

- In cases of high-grade serous carcinoma, ancillary techniques should be undertaken to help define the primary site.
- In cases of endometrioid EC a comment may be included on the unknown prognostic significance of this finding.

- Carcinomas metastatic to the wall of the fallopian tube and/or its serosa are viewed as metastatic from the endometrium, unless an origin in endometriosis is found, and the EC is accordingly upstaged.

Fallopian tube involvement in EC can have multiple possible sites/patterns. The following scenarios may be encountered:

1. *Fragments of tumor or aggregates of tumor cells within the tubal lumen, with no involvement of the mucosa, wall, or serosa*. In current practice, fragments of tumor and tumor cell aggregates are not uncommonly observed in the tubal lumen, mainly in patients operated on using techniques that involve an intrauterine manipulator, such as a laparoscopically assisted or robotic hysterectomy 45,46. So far, it has not been shown that these free-floating tumor fragments are associated with an adverse outcome; they are generally noted in the pathology report, but are not viewed as tubal involvement indicative of stage IIIA disease. This statement pertains to endometrioid EC, but the situation may be different if the EC is of serous type. Snyder et al. 47 reported that the presence of serous tumor cells in the tubal lumen was often associated with peritoneal metastases. Interestingly, it has been recently reported that patients with aggressive forms of EC such as serous carcinoma were likely to have lower stage disease and lower mortality if they had a prior tubal ligation, which would obstruct transtubal spread of tumor 48,49. Thus, it is particularly important to note the presence of intratubal tumor cells in serous carcinoma and other types of high-grade EC, although there is currently no consensus to upstage these EC based only on the presence of tumor cells within the fallopian tube lumen.
2. *Involvement of tubal mucosa only with a pattern of growth that raises the question of intraepithelial carcinoma*. Even though an intraepithelial carcinoma might be viewed as evidence of a primary process, it has been shown that metastatic carcinomas in the fallopian tube can show intraepithelial growth that mimics a primary intraepithelial carcinoma 50,51. Factors such as prior tubal ligation, site of tubal involvement, patterns of WT1, p53, and p16 immunohistochemistry in serous carcinomas and molecular studies may permit a more accurate determination of whether an intraepithelial carcinoma in the fallopian tube represents the primary site or, in rare cases, a synchronous primary 52,53. The distinction between so-called synchronous independent tubal and ECs and metastatic disease from 1 site to the other is discussed in more detail in another review in this issue 44.
3. *Involvement of mucosa and deeper layers of the fallopian tube wall, raising the differential diagnosis of synchronous primary tubal carcinoma versus metastasis*. The same principles as described above for intraepithelial involvement are used to determine the relationship between the neoplasms. It should be noted that detailed sampling of the fallopian tube, as has become commonplace in recent years, may result in increased detection of tubal mucosal spread in low-grade endometrioid EC, the prognostic significance of which is currently uncertain.
4. *Involvement of deep layers of the fallopian tube wall, often with tumor in lymphovascular spaces, with no mucosal involvement*. This is indicative of metastatic EC. A tubal carcinoma cannot be a synchronous primary tumor without a component that involves the tubal mucosa (including intraepithelial), so if the carcinoma is limited to the deeper layers of the fallopian tube wall or serosa, it is metastatic. The 1 exception to this rule could be a carcinoma that has arisen in tubal endometriosis but this is an extremely rare scenario.

LVSI can be seen in both primary and metastatic tubal tumors, but its presence should always result in the pathologist considering the possibility of metastasis. Occasionally, the only site of tubal involvement by a metastatic EC is within tubal lymphovascular spaces; an entirely intravascular carcinoma is certainly metastatic to the tube, but does not upstage the primary tumor. The prognostic significance of this finding has not been adequately studied.

Ovarian Involvement—Recommendations:

- Ovarian metastases in patients with EC should be categorized as stage IIIA.
- Ovarian carcinomas that are low-grade endometrioid in type and do not show features suggestive of metastasis, can be considered for staging and management as synchronous primary low-stage neoplasms, especially if associated with endometriosis. Such cases may be discussed at tumor board/multidisciplinary meetings.
- In the case of high-grade serous carcinoma involving the endometrium and ovary, most are ovarian tumors metastatic from the endometrial primary and this should result in tumor upstaging.
- In the extremely rare scenario where the ovarian tumor, if serous, is viewed as separate from the endometrial tumor based on immunohistochemical or molecular studies, it is important that the fallopian tubes be completely evaluated using the SEE-FIM protocol to exclude a tubal primary site.

The finding of tumor in the endometrium and ovary raises the differential diagnosis of synchronous primary tumors versus a metastasis, usually from the endometrium to the ovary. The distinction between so-called synchronous independent ovarian and ECs and metastatic disease from 1 site to the other is discussed in more detail in another review in this issue 44.

Involvement of Other Adnexal Sites, Such as Periadnexal Soft Tissue and Broad Ligament—Recommendation:

- Unless an obvious source for a primary carcinoma is identified, carcinomas in this region should be considered metastatic and the EC upstaged to IIIA.

Metastatic EC involving adnexal sites other than the ovary or fallopian tube has not been well studied. It is uncommon as an isolated finding, and when detected tends to occur in patients with tumor involvement of the fallopian tube or ovary. Pathologists always need to keep in mind that the same types of carcinoma that occur in the endometrium can rarely arise in the periadnexal soft tissue and broad ligament, from endometriosis, endosalpingiosis, or from benign tumors that occur at these sites, so the possibility of synchronous neoplasms does exist, although this is rare.

### Parametrial Invasion

Recommendations:

- Presence or absence of parametrial invasion should be recorded as its presence upstages an EC to stage IIIB.
- The fibroconnective tissue around the isthmus, at the cervix/lower uterine segment junction should be regarded as part of the parametria.
- Parametrial spread may be in direct continuity with the primary tumor (continuous spread), or may be identified as discrete metastases, in lymphovascular spaces, or in LNs (discontinuous spread).
- Continuous spread or discrete metastases into or in the parametrial tissue is required for diagnosis of parametrial involvement and staging of an EC as IIIB.

The parametria are composed of fibroconnective tissue, which surrounds the supravaginal part of the cervix and separates this part of the cervix anteriorly from the bladder and posteriorly from the rectum. This parametrial tissue extends onto the sides of the supravaginal cervix and between the layers of the broad ligaments 16. LNs, uterine blood vessels, and lymphatics that supply and drain the cervix are contained within the parametrial tissue. The ureters course downward and forward through the parametria on both sides of the cervix.

Radical hysterectomy includes a bilateral parametrectomy; this is generally performed for the treatment of cervical carcinoma and may be undertaken for ECs that arise in the lower uterine segment and/or secondarily extend to the cervix, in order to obtain clear surgical margins 2,19,54. Although a simple hysterectomy specimen, usually performed for ECs, includes minimal parametrial tissue, a small amount of fibrovascular connective tissue is usually attached 55 as the paracorpus and paracervix. Some pathologists embed the soft-tissue shavings from these areas and record whether they contain tumor or show LVSI but this is not uniform practice, nor is it evidence based.

Parametrial involvement by EC may be continuous or discontinuous from the main tumor. Discontinuous parametrial involvement may be seen as metastasis to LN, tumor within lymphovascular spaces, or as discrete metastatic deposits. Discontinuous infiltration of the parametrial soft tissue 19 as a result of outgrowth of tumor from foci of lymphovascular invasion is very rare. Parametrial involvement by EC typically occurs by direct extension of a deeply myoinvasive tumor or via LVSI. In either scenario, tumor carryover in the parametrium should be highly suspected in an EC without deep myometrial invasion or LVSI.

Stromal infiltration by EC is required to fulfill the criteria of parametrial involvement and thereby FIGO stage IIIB. The presence of tumor in lymphovascular spaces should be noted but does not upstage to stage IIIB; similarly the presence of parametrial LN involvement contributes to the overall LN staging, but does not constitute parametrial involvement. Continuous and discontinuous parametrial stromal infiltration are both staged as FIGO IIIB. Although the long-term prognosis is significantly poorer for endometrial cancer patients with parametrial spread, multivariate analysis at least in some studies has shown this parameter not to be an independent prognostic factor in EC 56.

### Vaginal Involvement

Recommendations:

- Presence or absence of vaginal involvement should be recorded where vaginal tissue is included in the resection specimen or submitted separately, as its presence upstages an EC to stage IIIB.
- The term “drop-metastasis” should not be used in surgical pathology reports, as it is nonspecific.
- Identification of any true involvement by EC (i.e. nonfloater), regardless of size, should be reported.

Vaginal involvement is currently defined in staging systems as present/absent 15, and the pattern of involvement is not further described. Vaginal involvement can be in the form of direct extension or “drop-metastasis,” the latter defined as tumor involvement without direct communication to the dominant mass and believed to arise from tumor seeding possibly as a result of surgical intervention. There is no minimum size criterion for vaginal involvement (whether considered to be direct extension or drop-metastasis). Similarly, the location of the metastasis (upper, middle, or lower third of vagina) plays no role in staging and does not appear to have an effect on patient outcome.

### LN Involvement (Nonsentinel)

Recommendations:

- Presence or absence of lymph nodal metastasis >0.2 mm in size should be recorded where LN are included as part of the resection as its presence upstages an EC to stage IIIC.
- Slicing of LN at 2 to 3 mm intervals along an axis perpendicular to the longest axis for histologic processing is preferable to bisecting nodes. If sectioned in this way, additional levels are not routinely recommended.
- The number of involved nodes and the total number of nodes retrieved from each site should be provided in the pathology report; in formal LN dissections, this may be expressed as the lymph node ratio (LNR) if this is the local reporting protocol.
- On the basis of limited evidence, the pattern of LN metastases and the presence of extracapsular spread should be reported.
- Size of metastatic nodal deposits may be documented in pathology reports.
- According to TNM 8, the presence of isolated tumor cells (ITCs) defined as metastatic disease <0.2 mm or pN0(i+) does not upstage an EC 14.

Nodal involvement is one of the most powerful prognostic determinants in all cancers, and predicts distant recurrence in low-risk EC 37. Pelvic (including parametrial) node involvement is FIGO stage IIIC1 and paraaortic nodal involvement stage IIIC2. The presence of positive nodes identifies a high-risk population and helps to tailor adjuvant treatment 57. However, at the same time, systematic pelvic (+/− paraaortic) nodal dissection, which is associated with significant morbidity, has not demonstrated any overall survival benefit 58,59, and thus practices vary worldwide. While in North America, there has been a high frequency of systematic node dissection, the extent to which routine lymphadenectomy is undertaken in the rest of the world varies considerably. It is important for standard and uniform protocols to be followed in LN sampling and reporting.

A further issue is the increased morbidity of nodal dissection carried out as a second procedure 60, which has prompted evaluation of intraoperative assessment and complete dissection as a single procedure; this is likely to vary with local practices, but, if offered, frozen section shows improved accuracy over imprint cytology for the detection of macrometastases, while both procedures miss micrometastases at the same frequency 61.

The major lymphatic trunks draining the uterus are uteroovarian (infundibulopelvic), parametrial, and presacral trunks, which drain into the hypogastric, external iliac, common iliac, presacral, and paraaortic nodes 62. To date, recommendations regarding LN counts in EC have not been reflective of the probability of finding disease. The Gynecologic Oncology Group Surgical Procedures Manual suggests that a minimum of 10 LNs be retrieved for evaluation. This is based on their recommendation that at least 1 LN be removed from each node-bearing region in the pelvis and paraaortic area 62,63. Recently, several studies have directly or indirectly examined the relationship between LN counts and the probability of finding nodal metastases in EC. Two retrospective reviews found that patients had improved survival when at least 10 to 12 LN were removed during lymphadenectomy. The improved survival was possibly due to adjuvant treatment following stage migration (better identification of patients with stage IIIC). Of note, paraaortic nodes may be positive in the absence of pelvic LN involvement in 9% of cases 64–66.

Study of deeper sections of conventional (nonsentinel) LNs has no value provided the LNs are sectioned at 2 to 3 mm intervals along an axis perpendicular to the longest axis (bread-sliced) rather than bisected 67, ideally with no >3 pieces per tissue cassette, as bisection presents less of the LN cross-sectional area than bread-slicing.

There are limited studies that correlate size/pattern of metastatic deposits in LNs with prognosis in EC. The ratio of positive to negative LNs or LNR has been investigated with a reduced progression-free survival in patients who had at least 10 LNs removed, and who had a LNR >50% compared with patients who had LNR ≤50% 68. A retrospective review of 612 surgically staged EC patients specifically looking at patterns of pelvic nodal involvement found that distant and paraaortic nodal recurrence was associated with extracapsular pelvic nodal disease as well as with metastases with a diameter >2 mm 69. A separate study also found the number of positive LN, desmoplasia in LNs, and extension of carcinoma into perinodal adipose tissue to be the most important adverse prognostic factors in stage IIIC EC 63.

### Sentinel LN Ultrastaging

Recommendations:

- Sentinel LNs may undergo ultrastaging by a protocol that includes examination of deeper levels +/− immunohistochemistry in addition to an initial hemotoxylin and eosin (H&E) section if this is negative for metastasis.
- Micrometastasis (defined as a focus of metastatic disease 0.2–2 mm in maximum diameter) and ITCs (defined as metastatic disease <0.2 mm) should be documented in the surgical pathology report, though the prognostic significance of these findings is unclear at present.
- Presence of ITCs does not upstage an EC 14.

Most ECs are confined to the uterus (FIGO stage I) and have 5-yr overall survival rates of 80% to 90%. However, about 10% to 15% of these patients will, in fact, have metastatic nodal disease, of whom nearly 15% have grade 1 endometrioid EC on preoperative biopsy; therefore, it is important to stage and treat these patients properly and avoid missing undetected metastatic disease.

Sentinel LN assessment is a useful option for these patients. For this reason sentinel node procedures have been evaluated in large series as well as randomized trial settings following breast cancer protocols, as an alternative to systematic node dissection 70–72. Sentinel node removal has been shown to be a safe alternative to LN dissection and long-term outcomes are now being reported with potential for development of optimal protocols in the near future 72,73. Sentinel node examination with ultrastaging may upstage low-risk and intermediate-risk EC 71, as well as high-risk EC 74. The presence of macrometastases (>2 mm) predicts involvement of nonsentinel nodes in EC, and is associated with worse outcome in comparison with node-negative EC 75. The long-term significance of ITCs (<0.2 mm) and micrometastases (0.2–2 mm) remains uncertain, as patients showing this very limited LN involvement have no significant differences in survival from patients with node-negative EC; this may be because these cases have often been treated as stage IIIC disease and have been given adjuvant therapy 70,72,73. Ultrastaging utilizing serial sections and cytokeratin stains improves detection of metastasis in individual studies, though this has not been confirmed in a recent metaanalysis 76. The above studies, that have reported long-term outcomes of low volume nodal metastases, state that further research is needed.

Several different protocols have been published for sentinel node ultrastaging, and all perform comparably with regard to detection of metastasis 70,77. Comparison of a comprehensive ultrastaging protocol with a simpler one showed that there was no statistically significant difference with respect to number of positive sentinel nodes detected, size of metastasis or false-negative rate 77. The presence of ITC does not upstage EC (TNM 8) 14.

Micrometastasis (defined as a focus of metastatic disease 0.2–2 mm in maximum diameter) and ITCs (defined as metastatic disease <0.2 mm) should be documented in the surgical pathology report, though the prognostic significance of these findings is unclear at present.

Confounding factors in the evaluation of metastases are the presence of mesothelial cells or benign epithelial inclusions (endosalpingiosis, endometriosis) within nodes. If mesothelial cells are suspected, staining for a mesothelial marker such as calretinin may be of value. Epithelial inclusion glands are typically seen within the LN capsule or septa and if necessary findings on immunostained slides should be correlated with H&E appearances.

### LVSI

Recommendations:

- Presence or absence of LVSI should be recorded in the pathology report.
- The sites of LVSI should be recorded.
- Before diagnosing LVSI, mimics should be excluded, such as retraction, MELF pattern of invasion, and artifactual displacement of tumor cells
- Immunohistochemistry is of limited use in the identification of LVSI.
- When present, LVSI should be reported as the number of vessels involved or semiquantified as “focal” or “substantial/extensive”.

**FIG. 6:**
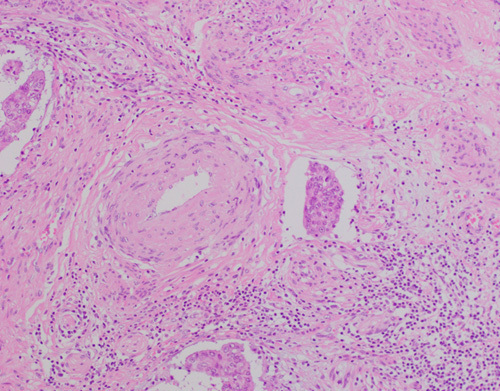
Lymphovascular space invasion in endometrial carcinoma.

LVSI is defined as the presence of viable tumor within endothelium-lined spaces, typically as clusters of cells that appear “free-floating” and often conform to the shape of the space (Fig. 6). The presence of associated proteinaceous material is helpful in recognition as a lymphatic space. In some studies, the finding of perivascular lymphocytic infiltrates has been shown to be associated with LVSI, but this finding is not diagnostic, and the presence of actual tumor emboli within the vessels is required for a diagnosis of LVSI 78,79.

The extent of LVSI is a prognostic factor. Involvement of more myometrial vessels, and vessels distant from the invasive front of the tumor predict LN metastasis and survival 80. Conversely LVSI confined to rare vessels involved at the invading front are of questionable prognostic value and classified by some pathologists as “indeterminate,” corresponding to the definition of “focal” below. A pooled analysis from 2 large clinical trials which included specialist review of 926 endometrioid EC for the presence of LVSI and evaluation of 2-tier, 3-tier, and 4-tier grading systems for LVSI has reported that: (a) the incidence of LVSI is low in stage I endometrioid EC (13.9% in this study); and (b) “substantial” LVSI, as opposed to “no” or “focal” LVSI, is the strongest independent prognostic factor for pelvic regional recurrence, distant recurrence, and overall survival 81. The definition of “substantial” in this study as the presence of ≥3 distinct, that is, widely separated vessels, is broadly in agreement with that of a consensus among gynecologic pathologists from different institutions, as absent, low (<3 vessel involvement), or extensive (≥3 vessels involved) 78. While of uncertain significance, it is suggested that the location (eg, deep myometrial, cervical, adnexal, parametrial, etc.) of LVSI be documented in the pathology report to allow for future studies to assess for possible significance.

Tumor cells can be displaced into vascular spaces during processing or uterine manipulation. Features of intravascular tumor morphology associated with so-called “vascular pseudoinvasion” include associated necroinflammatory debris or benign endometrial glands, or disaggregated tumor cells 79. Another artifact is the presence of tumor cells lodged in artifactual tissue clefts of the myometrium, which suggests a fragile tumor prone to displacement 45. Cases with these features should be evaluated carefully to distinguish between true LVSI and “vascular pseudoinvasion” and the 2 may coexist. In some cases, the distinction may be impossible and such cases should be classified as “indeterminate.” As the incidence of LVSI is low in low-stage endometrioid EC, an effort should be made to distinguish true LVSI from such mimics 79. Immunohistochemistry is of limited value in this distinction and reliance should be placed on the H&E appearance 82,83.

### Prognostic Factors Unrelated to Stage and With Limited/Conflicting Supporting Evidence

Recommendations:

- Tumor size should be included in the pathology report; this may be derived from macroscopic or, in the case of very small tumors, histologic assessment, or a combination.
- Tumor location may be included. In particular tumors located in the LUS region should be carefully evaluated as these may require distinction between an endocervical and an endometrial origin. Lower uterine segment ECs may be associated with Lynch Syndrome 84.
- Measurement of absolute depth of myometrial invasion, percentage of myometrium infiltrated by tumor, invasion of inner, middle, or outer one third of the myometrium, distance of myoinvasive tumor to serosal surface: any of these measurements may be carried out and included in the report according to local preference.
- Presence of a MELF pattern of myometrial invasion and presence or absence of histiocyte-like malignant cells may be recorded; although its prognostic significance has not been consistently demonstrated, the presence of this pattern of invasion warrants careful assessment of LVSI and avoidance of underestimation of myoinvasive depth.
- Peritoneal cytology is no longer necessary for staging but positive cytology should be recorded.

*Tumor size* is reported to predict LN involvement and recurrence and thereby can be utilized during preoperative or intraoperative assessment to determine the need for nodal dissection 37,85. Recording of tumor size is currently optional and is included in most reporting data sets as a noncore item, however, in incompletely staged apparent low-stage tumors, tumor size influences the recommendation for adjuvant therapy 1.

*Tumor location* within the uterine corpus may have clinical significance and should be recorded as LUS, body, fundus, or cornu. Approximately 14% of ECs arise in the lower uterine segment and these are more frequently associated with mismatch repair gene abnormalities and Lynch Syndrome. Lower uterine segment involvement has also been shown to be an independent prognostic factor in conferring a higher risk of distant recurrence and death 84,86. Furthermore, when a tumor involves the lower uterine segment without a tumor mass in the corpus, it is important to distinguish between a cervical and an endometrial origin, as this has important management implications (this distinction is discussed in more detail in another review in this issue 44).

*Measurement of absolute depth of myometrial invasion, invasion of inner, middle, or outer one third of the myometrium, percentage of myometrium infiltrated by tumor, distance of myoinvasive tumor to serosal surface.* These related absolute measurements (in mm) have been studied and not found to be consistently and independently predictive of prognosis. These do not add to the categorical assessment of inner half versus half or more of myometrial thickness involvement, which is also integral to tumor staging. Any of these may be included in the report but are currently optional and included in most reporting data sets as noncore items.

*MELF pattern* of myometrial invasion is reported to correlate with lymph nodal metastasis 87,88. In addition to conventional expansile and infiltrative patterns of myometrial invasion 89, rarer patterns of invasion may occur in endometrioid EC. These include an adenoma malignum-like pattern and MELF-type invasion. The latter is characterized by a prominent fibromyxoid desmoplastic stromal reaction, with a subset showing outpouchings and detached glands which are dilated and lined by flattened epithelium creating microcysts. There may also be gland elongation or fragmentation resulting in small solid clusters of cells or single cells. This pattern of myoinvasion shows immunohistochemical features in tumor cells suggesting that the phenotypic changes are related to the phenomenon of “epithelial-mesenchymal transition” 30,90. A MELF pattern of invasion is reported to be associated with a higher likelihood of nodal involvement and distant recurrence but its significance as an independent risk factor remains unclear 91,92, possibly partly related to a level of subjectivity in its recognition.

**FIG. 7:**
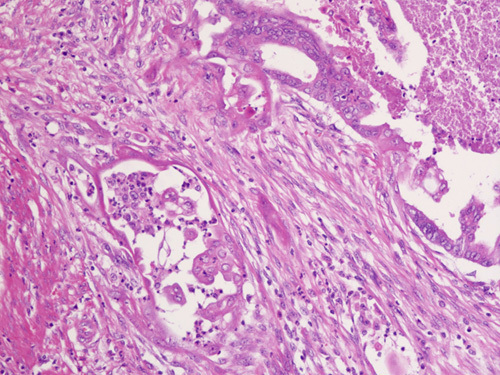
Tumor cells in areas with a microcystic, elongated, and fragmented pattern of invasion appear different from the main tumor; in comparison to the columnar epithelium in glands in the upper right area, the glands on the left are lined by cells that are paler, flatter, and show a histiocytoid appearance when individually dispersed.

**FIG. 8:**
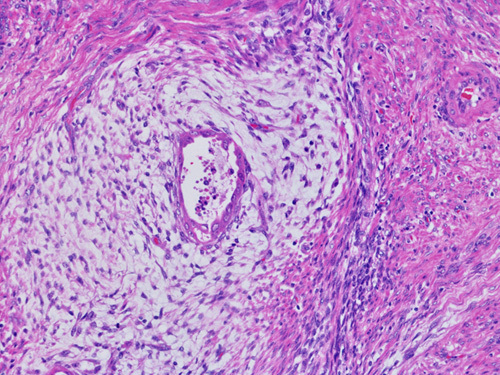
A conspicuous edematous and fibromyxoid zone surrounds infiltrative glands in areas with a microcystic, elongated, and fragmented pattern; for the purposes of measuring myoinvasive depth it is the epithelial element and not the stromal reaction that is taken into account.

Therefore, the presence of MELF-pattern invasion does not currently influence patient management but together with other invasive patterns remains the focus of research studies. Nevertheless, the recognition and recording of this parameter is important for other reasons. First, this is a subtle pattern often seen focally at the invasive front in tumors that are low grade (Fig. 7). Because of the relative pallor and different appearance of the tumor at these sites, it may be missed, resulting in underestimation of depth of invasion. Second, this is typically seen as glands with a markedly attenuated lining showing detached tumor cells within the lumen, findings that may be wrongly interpreted as LVSI. Third, this pattern is significantly associated with LVSI, and when present a careful search should be performed to exclude as well as correctly identify LVSI, which is otherwise an uncommon finding in low-grade endometrioid EC. Fourth, the presence of MELF-pattern invasion will prompt histopathologic ultrastaging of resected LNs for occult metastatic involvement within some institutions. Finally, as discussed above in the section on “myometrial invasion,” the depth of invasion in this and other ECs with a fibromyxoid stromal reaction, should be based on the finding of neoplastic epithelial cells, and not the reactive stroma (Fig. 8).

### Peritoneal Cytology

Positive peritoneal cytology is no longer included in staging of EC but is recommended as part of the surgical procedure 1 (National Comprehensive Cancer Network). There is lack of consensus in the literature regarding its prognostic significance in the absence of other evidence of extrauterine spread. A recommendation has been made by FIGO and the Union for International Cancer Control to record the status of peritoneal washings if these have been carried out, without altering the tumor stage, if these are positive for malignant cells 15, as this is taken to be an adverse risk factor when considering adjuvant therapy 1.

## CONCLUSIONS

A number of factors related to and independent of tumor stage help to determine the need for adjuvant therapies in patients with EC. The accurate and consistent reporting of these pathologic parameters is vital to ensure optimal patient management.
